# Factors Related to Oversleeping in Korean Young Adults, with a Focus on Sociodemographic Factors

**DOI:** 10.3390/ijerph191710485

**Published:** 2022-08-23

**Authors:** Jin-Won Noh, Jinseok Kim, Yejin Lee, Young Dae Kwon

**Affiliations:** 1 Division of Health Administration, College of Software and Digital Healthcare Convergence, Yonsei University, Wonju 26493, Korea; 2 Department of Social Welfare, Seoul Women’s University, Seoul 01797, Korea; 3 Department of Public Health, Graduate School, Korea University, Seoul 02841, Korea; 4 Department of Humanities and Social Medicine, College of Medicine, Catholic Institute for Healthcare Management, The Catholic University of Korea, Seoul 06591, Korea

**Keywords:** oversleeping, young adults, sociodemographic factors, long sleep duration

## Abstract

Young adults the year after high school experience changes in lifestyle and circumstances and tend to experience more oversleeping. However, there are few studies on oversleeping in young adults. This study aims to identify the sociodemographic factors related to oversleeping among young adults in Korea using nationally representative data. This study analyzed self-reported and cross-sectional data in 2016 from a sample of 1876 participants from the Korean Children and Youth Panel Survey, which included respondents one year after high school graduation. Oversleeping was defined as sleeping 9 or more hours a day on average. Logistic regression models were analyzed to test a multivariate association between independent sociodemographic variables and oversleeping. Over one-tenth of young adults reported oversleeping. Oversleeping was associated with household income (OR = 0.99, *p* = 0.011) and smoking (OR = 1.52, *p* = 0.041). In addition, when compared to non-working college students, college students who were working (OR = 2.58, *p* = 0.021), non-students who were working (OR = 1.68, *p* = 0.048), and non-students who were not working (OR = 3.07, *p* < 0.001) were more likely to report oversleeping. Oversleeping among young adults was associated with major sociodemographic factors including household income (−), smoking (+), and academic and working status (+). These findings suggest the significant role of sociodemographic factors as predictors of oversleeping and emphasize the importance of examining various factors to achieve a better understanding of oversleeping in young adults.

## 1. Introduction

Young adulthood, between the ages of 18 and 25, is defined as a unique period of rapid physical, physiological, neurological, and behavioral changes that begin with puberty and end in adulthood [1,2]. Young adulthood lays the foundation for taking on adult roles and responsibilities, such as transitioning to employment and financial independence and forming life partnerships [2,3]. Young adults require sufficient sleep to function normally and maintain physical, emotional, and cognitive health [4,5]. Young adults are recommended to get seven to nine hours of sleep by the US National Sleep Foundation [6]. However, they often experience sleep problems, including insufficient sleep and oversleeping [7]. Previous studies on sleep problems in adolescents have primarily focused on insufficient sleep and short sleep duration [4,5,8,9,10]. There are limited studies on oversleeping [11,12].

These sleep problems in young adults are believed to be influenced by psychosocial factors such as increasing peer pressure to stay up late and a co-occurring reduction of parental influence on young adult sleep patterns [7]. Oversleeping and compensating behavior such as napping or sleeping in on weekends may further contribute to the sleep/wake rhythm delay, making it difficult to fall asleep at appropriate times. By this, some young adults perceive themselves as being trapped in a vicious cycle or positive feedback loop [7]. Under those circumstances, a previous study reported that weekend oversleeping, both short and long durations, was associated with psychological disorders, tobacco smoking, and poor perceived mental and physical health [13]. Another previous study reported that oversleeping can be used to identify negative physical health patterns, including atypical depression, increased distress, and disability [14]. In addition, oversleeping leads to poor academic performance [15].

From a developmental point of view in the life cycle, young adults face important evolutionary tasks (i.e., the acquisition of identity, autonomy, and self-determination; the reorganization of relationships with parents and peers) [16] and experience a critical period marked by instability, anxiety, and depression [17]. Young adults are reported to have high rates of injuries, psychological health issues, substance abuse, and sexual/reproductive health problems. Young adults are in a vulnerable state due to higher risk-taking behavior, which is relatively associated with poor health outcomes [1]. Thus, it can be a period of higher risk for the onset of psychopathological problems [18,19], and these life cycle characteristics of young adulthood can aggravate sleep problems, including oversleeping [16].

In particular, many previous studies reported that Korean young adults are suffering from sleep problems due to excessive academic pressure and the competitive college entrance examination [20,21]. Most previous research has focused on short sleep duration. However, young adults face different social expectations and demands different from adolescents [22]. College students usually face numerous challenges, such as different types of academic pressures, social obligations, Internet distraction, self-reliance, and irregular schedules [23]. Previous studies reported that college students tend to oversleep due to increased academic demand and social relationships [24]. However, there are few studies on oversleeping and its related factors in young adults. Thus, this study aims to identify the incidence of oversleeping and the sociodemographic factors related to oversleeping in Korean young adults, especially in the year after high school, using nationally representative data.

## 2. Materials and Methods

### 2.1. Data and Subjects

**Figure 1:**
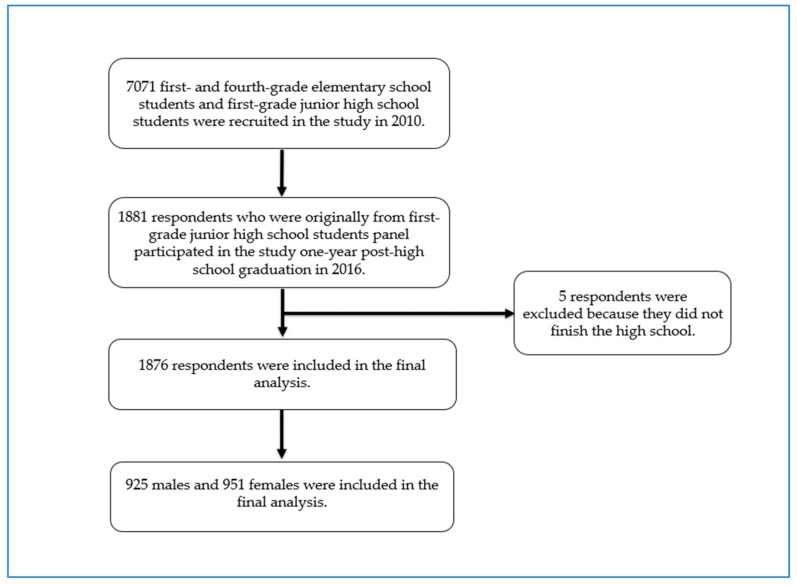
Selection process of study subjects.

Data from the Korean Children and Youth Panel Survey (KCYPS), a nationally representative study of Korean children and youths, were used for this study [25,26]. During the period between 2010 and 2016, the KCYPS collected data using respondents’ self-report from three different panels of first- and fourth-grade elementary school students and first-grade junior high school students (*n* = 7071) aiming to investigate various aspects of children’s and youths’ growth and development. A multi-stage stratified cluster sampling method with schools as the primary sampling unit was employed in the KCYPS. This analysis utilized the cross-sectional data from the last wave in 2016 of the junior high school student panel, which was when the respondents were one-year post-high school graduation. Among 1881 youths who participated in the last wave of this study, 5 participants who did not finish high school were excluded from this analysis because their life situation was deemed quite different from those who graduated from high school, which resulted in a final analysis sample consisting of 1876 participants (Figure 1).

This study was approved by the Institutional Review Board of the Seoul Women’s University (IRB-2018-46) with a waiver for informed consent because the data were obtained from a public data repository.

### 2.2. Variables and Measurements

Following the recommended sleep times by the US National Sleep Foundation of seven to nine hours a day for youths aged 18–25 years old, oversleeping was defined as sleeping 9 or more hours a day on average [6]. The American Academy of Sleep Medicine and Sleep Research Society has also recommended the appropriate amount of sleep needed for adults [27]. Sleep duration was calculated using bedtime and wake-up time, which were ascertained by using the question “What time do you usually go to bed and get up?” Because the sleep-related question was asked for weekdays and for weekend days separately, the average sleep duration was calculated as follows: [(weekday sleep duration) × 5 + (weekend sleep duration) × 2]/7. The oversleeping variable was coded as 1 if a respondent’s average sleep duration was 9 h (540 min) or longer and 0 otherwise.

Several sociodemographic variables and health risk behaviors such as smoking and alcohol consumption during the last year were included in this analysis. The health risk behaviors were measured using respondent self-reports of such behaviors. In addition, respondents’ screen time was defined as the total number of hours spent on television and video games and included in the analysis. Gender, household income, regional area, respondents’ academic and working status, parents’ working status, cohabiting status with respondents’ parents, and annual household income were included as independent variables. Household income, cohabiting status, and parents’ working status were asked to respondents’ parents or primary caretakers. Because the participants were from the same age cohort, age was not considered in the analysis.

Gender was measured using a dichotomous variable (male = 1, female = 0), and regional area was also dummy-coded (urban = 1, non-urban = 0). Finally, respondents’ college enrollment status and working status were combined to produce a variable comprising four different categories: non-working college student, working college student, working non-college student, and non-working non-college student.

### 2.3. Statistical Analysis

To provide sample characteristics, a set of descriptive analyses were conducted. We conducted a series of bivariate analysis such as the Chi-squared test of independence or the t-test to evaluate the association between oversleeping and other relevant variables. Finally, logistic regression models were analyzed to test a multivariate association between a set of independent variables and the oversleeping variable. Stata 17 (StataCorp LP, College Station, TX, USA) was used to manipulate the data and calculate the model parameters.

## 3. Results

The characteristics of the sample are presented in Table 1. Males (49.3%) and females (50.7%) were almost evenly included in this study. Almost two-thirds of mothers (65.2%) and 9 out of 10 (89.7%) fathers were working outside the home. Over half (58.3%) of the respondents reported that both parents were working. About two-thirds of young adults (65.5%) were living with their parents. A majority of the respondents (84.2%) were living in urban areas. About four out of five drank alcohol (80.1%), and a similar proportion of the respondents did not smoke cigarettes (79.8%). Respondents reported an average screen time per week and annual household income of 15.9 h and 10k KRW 4917.5, respectively. Over one-tenth (10.9%) of the respondents reported oversleeping behaviors.

Table 1 also presents the proportion of young adults who experienced oversleeping by various related factors, including gender, parents’ working status, whether living with parents, living area, and status of working and studying. Average hours of screen time per week are also detailed in Table 1, including video games and television, and household income based on the respondents’ oversleeping status. The results showed that young adults whose fathers were working were less likely to experience oversleeping than the same whose fathers were not working (Chi-squared (1) = 13.88, *p* < 0.001). Similarly, young adults whose parents were both working were less likely to experience oversleeping than those whose parents were both unemployed (Chi-squared (1) = 4.51, *p* = 0.034). Furthermore, young adults who were attending college and not working were less likely to experience oversleeping than those who were neither working nor attending college (Chi-squared (3) = 36.42, *p* < 0.001). Young adults who reported smoking behavior were more likely to experience oversleeping than those who did not (Chi-squared (1) = 8.50, *p* = 0.004). In addition, the household income of those who experienced oversleeping was lower than the same of those who did not (t = 3.83, *p* < 0.001) (Table 1).

Furthermore, the results from a multivariate logistic regression indicated that household income (OR (SE) = 0.99 (<0.01), *p* = 0.011) and smoking behavior (OR (SE) = 1.52 (0.31) *p* = 0.041) were associated with oversleeping behavior. In addition, when compared to non-working college students, college students who were working (OR (SE) = 2.58 (1.06), *p* = 0.021), non-students who were working (OR (SE) = 1.68 (0.44), *p* = 0.048), and non-students who were not working (OR (SE) = 3.07 (0.62) *p* < 0.001) were more likely to report oversleeping (Table 2).

## 4. Discussion

This study aimed to identify the incidence of oversleeping and the sociodemographic factors related to oversleeping in Korean young adults, especially in the year after high school, using nationally representative data. A previous study of US adolescents aged 12–17 years old in 2014 identified that 13.1% of adolescents reported oversleeping (sleeping more than 10 h on weekdays), using data from the National Cancer Institute’s Family Life, Activity, Sun, Health, and Eating Study [28]. Another previous study of individuals in 24 countries (21 European countries, Korea, Japan, and Thailand) aged 17–30 years old identified that 6% of respondents reported oversleeping (average sleep duration > 10 h), using data from the International Health and Behavior Study [29]. In the present study, 10.9% of the respondents reported oversleeping, which is higher than the results from the European and Asian countries and lower than the results for US adolescents. The year after high school is a period of transition for young adults during which they frequently experience oversleeping, compared to adolescents in high school [30].

This study identified that lower household income was associated with experiencing oversleep behavior. Socioeconomic status was regarded as an important factor affecting sleep duration in adolescents and young adults. A previous study of Korean adolescents reported that adolescents with higher-income families tend to sleep fewer hours per night [31]. In Korea, highly educated parents with high occupational statuses tend to obsess over prominent universities and have higher educational aspirations for their children than do less educated parents with low occupational statuses [32]. They tend to make large investments in private education, and their children arrive home later, which is linked to reduced sleep duration [33]. However, studies of Western countries often report contradictory results. A European study reported that adolescents’ sleep duration had no significant relationship with their parents’ socioeconomic status [33]. Other studies in Australia and New Zealand reported that adolescents from families with lower socioeconomic status tend to sleep shorter times [34,35]. The results in Western countries can be explained in terms of working time; the use of media, such as computer games and television in the bedroom; and insufficient sleep education, attributed to the parents’ lack of education [33].

In addition, this study indicated that smoking cigarettes was associated with experiencing oversleep behavior. Particularly, a previous study of national US young adults reported that about 40% of those in their first year after high school experienced binge drinking in the last 30 days (46% among college students), and over 20% experienced marijuana use in the last 30 days (22% among college students) [30]. These results might be attributed to the unique character of these young adults, substantial leisure time, or exposure to the leisure of work environments conducive to excess drinking [30]. Another previous study in the US reported that the past use of any illicit drug, including cigarettes and alcohol, is associated with sleep problems in young adults [36]. It has also been proven in other studies of adults that substance use (cigarettes, alcohol, and illicit drugs) is associated with sleep disturbances such as insomnia, hypersomnia, and parasomnia [37]. Several studies have found significant association between tobacco smoking and oversleeping. Daily smokers were more likely to have difficulty falling asleep than nonsmokers [38], and smoking cigarettes was associated with an increased risk of future sleep problems [39].

In this study, college students who were working, non-college students who were working, and non-college students who were not working were more likely to report oversleeping when compared to non-working college students. The year after high school is a period of transition for young adults as they attend college, work, and interact with new social and physical environments that can influence their health behaviors [30]. It is well-known that the stress in college students’ lives involves their pursuit of part-time or full-time employment during their undergraduate courses [40]. Although working during college may help college students learn time management skills and help cover the costs of their education [41], it not only increases the input of work hours associated with fewer sleep hours [42], but also increases the stress of balancing a busy schedule that can also lead to poorer sleep quality [41]. Previous studies indicated an inverse relationship between hours spent working and sleep duration [43,44]. Moreover, full-time employees tend to work longer hours and consequently sleep for a shorter period during the night [45]. Meanwhile, those who did not attend college reported bad health behaviors, such as drinking heavily, driving while intoxicated, and more physical and depressive symptoms. Thus, they reported experiencing more oversleeping [37]. The results of this study provide important implications that oversleeping in young adults has an association with their academic and working status.

This study has some limitations. First, only a single question was used to measure bedtime and wake-up time and calculate the sleep duration. The sleep duration difference between weekday and weekend was not considered separately, and all measurements of each variable were self-reported, which can increase potential response bias or recall bias. To test the presented model more thoroughly, future studies should supplement self-reported measurements with alternative methodologies (e.g., daily sleep diaries, actigraphy). In addition, further studies need to consider weighted average of weekday and weekend sleep duration. Second, this study only analyzed oversleeping using wake-up time and bedtime but did not consider sleep quality or other health problems that could affect sleep patterns among young adults. In addition, this study measured health risk behaviors, including alcohol consumption and cigarette smoking, by binary questionnaires (yes/no). Thus, future studies should evaluate oversleeping with other physical and psychological health problems of young adults, using various validated scales. Third, this study focused only on the presence or absence of oversleeping. In future studies, it would be good to compare the three groups including sleep deprivation. Lastly, these cross-sectional data do not allow us to analyze the temporal sequence between sociodemographic factors and oversleeping in young adults, which precludes any causal inference. Future studies should examine these associations longitudinally to better ascertain the direction of effects.

Despite these limitations, this study analyzed hitherto under-addressed sociodemographic factors associated with oversleeping in young adulthood which have been known as facing numerous challenges, using nationally representative data. This study provides implications that sociodemographic factors might have a key role to understand and improve oversleeping of young adults in a unique and diversified lifecycle. These findings could provide the basis for further studies to assess the importance and health outcomes of oversleeping in young adults.

## 5. Conclusions

The current study identified meaningful sociodemographic factors (i.e., academic and working status, household income, and smoking cigarettes) that are particularly associated with experiencing oversleeping in young adults, using nationally representative data. It provides implications that sociodemographic factors might have a fundamental role in the sleep schedule of young adults. Better socioeconomic status was regarded as an important factor in improving sleep in young adults, and these findings provide important information to policy makers to develop alternative options by addressing sociodemographic factors. Due to the unique characteristics and related health outcomes, young adulthood should be categorized and studied separately from adolescence and adulthood. Further research using longitudinal and representative data is needed to identify the causal inferences that lead to advanced oversleeping problems in young adults, considering their physical and psychological health.

## Figures and Tables

**Table 1:** Sample characteristics and proportion of Korean young adults who experienced oversleeping by various related factors (*n* = 1876).

Variable	Category	Total		Oversleep		Chi-Squared
		n	%	No (%)	Yes (%)	
Oversleeping	No	1672	89.1			
	Yes	204	10.9			
Gender	Male	925	49.3	88.5	11.5	0.64
	Female	951	50.7	89.7	10.3	
Mother working	No	659	34.8	88.0	12.0	1.10
	Yes	1237	65.2	89.7	10.4	
Father working	No	195	10.3	81.0	19.0	13.88 ***
	Yes	1701	89.7	90.0	10.0	
Both parents working	No	790	41.7	87.2	12.8	4.51 *
	Yes	1106	58.3	90.4	9.6	
Living with parents	No	647	34.5	89.8	10.2	0.46
	Yes	1229	65.5	88.8	11.2	
Living area	Rural area	300	15.8	88.4	11.6	0.16
	Urban area	1596	84.2	89.2	10.8	
Academic and working status	College student, not working	1287	68.6	91.9	8.1	36.42 ***
	College student, working	61	3.3	86.9	13.1	
	Not college student, working	197	10.5	85.3	14.7	
	Not college student, not working	331	17.6	81.0	19.0	
Alcohol consumption	No	374	19.9	88.5	11.5	0.19
	Yes	1502	80.1	89.3	10.7	
Cigarette smoking	No	1497	79.8	90.2	9.8	8.50 **
	Yes	379	20.2	85.0	15.0	
		**Mean**	**SD**	**Mean**	**Mean**	**t**
Screen time per week	(Wave 6 h)	15.9	12.5	15.8	17.1	1.37
Household income	(Annual income, KRW 10k)	4917.5	2569.8	5001.0	4235.6	3.83 ***

* *p* < 0.05; ** *p* < 0.01; *** *p* < 0.001; SD, standard deviation; KRW, Korean won.

**Table 2:** Logistic regression analysis to investigate factors associated with oversleeping (*n* = 1651).

Variable	Category	OR	SE	95% CI
Gender	Male ^a^			
	Female	1.09	0.19	(0.78, 1.54)
Mother working	No ^a^			
	Yes	0.87	0.41	(0.34, 2.19)
Father working	No ^a^			
	Yes	0.57	0.25	(0.24, 1.34)
Both parents working	No ^a^			
	Yes	1.23	0.62	(0.45, 3.33)
Living with parents	No ^a^			
	Yes	0.86	0.16	(0.60, 1.25)
Academic and working status	College student, not working ^a^			
	College student, working	2.58 *	1.06	(1.15, 5.77)
	Not college student, working	1.68 *	0.44	(1.00, 2.79)
	Not college student, not working	3.07 ***	0.62	(2.08, 4.55)
Living area	Rural ^a^			
	Urban	0.89	0.20	(0.58, 1.37)
Screen time per week ^b^	(Wave 6 h)	1.00	0.01	(0.99, 1.02)
Household income ^b^	(Annual income, million KRW)	0.99 *	0.00	(0.98, 1.00)
Alcohol consumption	No ^a^			
	Yes	0.82	0.17	(0.55, 1.23)
Cigarette smoking	No ^a^			
	Yes	1.52 *	0.31	(1.02, 2.26)
Intercept		0.23 *	0.13	(0.08, 0.71)
Pseudo R-squared = 0.054	LR chi-squared (df = 13) = 60.73 ***			

* *p* < 0.05; *** *p* < 0.001; ^a^ reference category; ^b^ continuous variable; OR, odds ratio; SE, standard error; CI, confidence interval; KRW, Korean won.

## Data Availability

The data presented in this study are openly available in NYPI Youth and Children Data Archive at https://www.nypi.re.kr/archive/board?menuId=MENU00329 (accessed on 7 February 2022).
